# Supplementary material for: Polyketide synthase gene clusters in the ericoid mycorrhizal fungus Oidiodendron maius: genome-wide prediction, expression profiling, and functional characterization of knockout mutants
Source: Front Plant Sci. 2026 Sep 16;17:1924430. doi: 10.3389/fpls.2026.1924430 (PMC13623903; doi:10.3389/fpls.2026.1924430)
Supplement: Supplementary file 1 [file SupplementaryFile1.zip › Supplementary Materials/Supplementary Folder 1.zip!/Oidma1_AssemblyScaffolds_Repeatmasked/knownclusterblast/cluster4/jgi.p_Oidma1_36694_mibig_hits.html]

| MIBiG Protein | Description | MIBiG Cluster | MiBiG Product | % ID | % Coverage | BLAST Score | E-value |
| --- | --- | --- | --- | --- | --- | --- | --- |
| EAU38781.1 | conserved\_hypothetical\_protein | BGC0000161 | Terrein\_biosynthetic\_gene\_cluster | 57 | 99.5689655172 | 549 | 3e-156 |
| EAU38784.1 | conserved\_hypothetical\_protein | BGC0000161 | Terrein\_biosynthetic\_gene\_cluster | 42 | 99.1379310345 | 358 | 1.3e-98 |
| AMK92567.1 | tetracenomycin\_c\_resistance\_and\_export\_protein | BGC0001377 | Uncialamycin\_biosynthetic\_gene\_cluster | 25 | 81.4655172414 | 90 | 6.5e-18 |
| AME18021.1 | transporter | BGC0001378 | Tiancimycin\_biosynthetic\_gene\_cluster | 24 | 81.0344827586 | 85 | 1.6e-16 |
| ACU71641.1 | drug\_resistance\_transporter,\_EmrB/QacA\_subfamily | BGC0001154 | Cacibiocin\_biosynthetic\_gene\_cluster | 24 | 84.0517241379 | 77 | 5.7e-14 |
| ADZ13560.1 | YtkR6 | BGC0000466 | Yatakemycin\_biosynthetic\_gene\_cluster | 23 | 83.6206896552 | 72 | 1.8e-12 |
| ACS13711.1 | PtmP4 | BGC0001140 | Platensimycin\_/\_platencin\_biosynthetic\_gene\_cluster | 21 | 83.4051724138 | 70 | 7e-12 |
| ADD83011.1 | PtnP4 | BGC0001156 | Platencin\_biosynthetic\_gene\_cluster | 21 | 83.6206896552 | 69 | 1.6e-11 |
